# On Secure Simple Pairing in Bluetooth Standard v5.0-Part I: Authenticated Link Key Security and Its Home Automation and Entertainment Applications

**DOI:** 10.3390/s19051158

**Published:** 2019-03-07

**Authors:** Da-Zhi Sun, Li Sun

**Affiliations:** Tianjin Key Laboratory of Advanced Networking (TANK), Division of Intelligence and Computing, Tianjin University, No. 135, Yaguan Road, Tianjin Haihe Education Park, Tianjin 300350, China; sunlibella@tju.edu.cn

**Keywords:** Bluetooth standard, Secure simple pairing, Cryptographic protocol, Security model, Authenticated link key, Home automation and entertainment

## Abstract

Bluetooth is an important technical standard for short-range and low-power wireless communication. The home automation and entertainment (HAE) systems often make use of Bluetooth technology to link different Bluetooth devices and form Bluetooth networks. The security concerns of the HAE systems are raised due to massive deployment of the Bluetooth devices. The Bluetooth standard mainly depends on the secure simple pairing (SSP) solution to protect the Bluetooth devices. Hence, we investigate the SSP solution according to the Bluetooth standard v5.0. The contributions are threefold. (1) A formal security model is proposed to evaluate SSP’s association models and authenticated link key. (2) We formally analyze two SSP protocols and present the security requirements for basic cryptographic modules in these SSP protocols. (3) We discuss the typical SSP applications in the HAE systems. Our results are useful to not only evaluating and designing the SSP protocols but also enhancing the security of the HAE systems in which the Bluetooth access is available.

## 1. Introduction

Owing to the rapid development of the Internet of Things (IoT), the home automation and entertainment (HAE) systems simplify the controls of different home appliances and enhance their convenience, safety, and comfort via either wired or wireless communication. The idea of HAE emphasizes that multiple home appliances could be controlled by a single controller via a home network. We see that Bluetooth [1] always provides the private wireless connections and the confidential data transmissions among home appliances because the Bluetooth services maintain ubiquity, reliability, and interoperability. In fact, how to integrate Bluetooth into the HAE environments has drawn a great deal of attention and become a hot topic in the research community. However, the Bluetooth devices may leak confidential data and the adversary may monitor the Bluetooth channel during the communication procedure. Hence, Bluetooth security is very important when the Bluetooth services process sensitive information in the HAE systems.

According to latest Bluetooth standard v5.0 [2,3], it provides five different security features. That is, pairing, bonding, device authentication, encryption, and message integrity. We outline as follows.

- Pairing: Establishing shared keys among Bluetooth devices paired.
- Bonding: Establishing a trusted bonding relationship between two Bluetooth devices relying on pairing.
- Authentication: Verifying that the same key exists between two Bluetooth devices.
- Encryption: Keeping messages confidential.
- Message integrity: Verifying that messages are not forged.

From the technical perspective, pairing is the first and important step to ensure Bluetooth security, because the function of pairing is to establish a shared link key and the link key is the master key for other Bluetooth mechanisms. Currently, Bluetooth standard v5.0 [2,3] preferably supports secure simple pairing (SSP) to provide the function of pairing. SSP can prevent passive eavesdropping and man-in-the-middle (MITM) attacks. There are four association models of SSP depending on IO capabilities of devices, i.e., numeric comparison (NC), out of band (OOB), passkey entry (PE), and just works (JW). SSP must use one of these association models to complete the pairing procedure. Actually, four association models form four different SSP protocols, i.e., the NC protocol, the OOB protocol, the PE protocol, and the JW protocol.

The main goal of our research is to address the security and the privacy of SSP in Bluetooth standard v5.0. This paper is part one, which focuses on the security of SSP and its HAE applications. We formally address the security of the authenticated link key generated by SSP. That is, we mainly focus on the security of the NC and OOB protocols. Some result of the PE protocol is presented directly. We do not specially discuss the JW protocol, because the JW protocol is the same as the NC protocol except that it does not defeat MITM attacks. We also investigate how to integrate the typical SSP applications into the HAE systems. Part two of our research will focus on the privacy issue of SSP in Bluetooth standard v5.0.

### 1.1. Related Work

Much attention has been devoted to the SSP security in four association models along with a wide utilization in Bluetooth technology. Lindell [4] proposed that the PE protocol would leak password by eavesdropping attack on a legitimate conversation or an active attack on password-protected devices, which may cause MITM attacks. Haataja et al. [5,6] proposed MITM attacks might be implemented as follows. (1) The adversary forges information during IO capabilities exchange and forces device to use the JW protocol. Padgette et al. [7] also discussed this vulnerability. (2) Legitimate users are misled to select a less secure option instead of using a more secure OOB channel. Sandhya et al. [8] and Phan et al. [9] all explored basic security properties required by SSP, i.e., known key security, key control, perfect forward secrecy, key-compromise impersonation, unknown key-share attack resilience, MITM attack resilience, and offline dictionary attack resilience. Barnickel et al. [10] showed that the PE protocol leaks passkey to any adversary eavesdropping and is vulnerable to MITM attacks if the user uses the same passkey twice (or a fixed passkey is used). Albahar et al. [11] enhanced the JW protocol to prevent MITM attacks by adding a question and answer phase to ask both Bluetooth devices about IO capabilities. Gajbhiye et al. [12,13] overviewed security issues that may result in MITM attacks on Bluetooth devices and improved SSP with the delayed-encrypted input-output mechanism to defeat MITM attacks. In addition, they also gave the simulation and the security analysis of the NC protocol. Sun et al. [14] showed that the PE protocol is vulnerable to MITM attacks, once the host reuses the passkey. Lonzetta et al. [15] examined the Bluetooth’s risks in the smart homes and their countermeasures, where SSP is suggested to take place of legacy personal identification number (PIN) authentication to avoid PIN cracking attacks.

Despite a great deal of analysis of SSP’s vulnerabilities and countermeasures up to now, there is little formal analysis of SSP in different association models. Bellare et al.’s formal works [16,17,18,19,20,21] gave us a great inspiration to formally prove the security of the SSP protocols. Formal verifications of the NC protocol and the OOB protocol using ProVerif were proposed in [22]. Yeh et al. [23] presented the weaknesses of SSP, that is, the adversary could exploit the public keys exchanged in SSP protocol to carry out MITM attacks because the public keys are not authenticated. Benin et al. [24] addressed secure association for devices whose association process is vulnerable to MITM attacks and formally proved that their new message recognition protocol satisfies their security definition. Lindell [25] presented a rigorous proof that the NC protocol for device pairing in Bluetooth standard v2.1 is secure of non-triviality, correctness, and privacy.

Customers raise security concerns due to the massive deployment of the Bluetooth HAE systems. Health monitoring system [26] needs to transfer private health data. Wearables [27,28] store and share many sensitive data. Bluetooth smart analyzer [29] is related to users’ indoor location. Home smart lock systems [30,31,32,33,34] provide access to the door only for the authorized users. IoT-based emergency and disaster relief system [35] needs to share private data to different service providers. Voice operated room automation system [36] involves both connection of personal devices and transmission of private data. Bluetooth-based indoor localization systems [37,38,39] must prevent forgery and substitution attack by malicious users. All above HAE systems adopt the Bluetooth services to provide transmission and storage functions for sensitive information. Hence, it is indispensable to provide the rigorous analysis of the SSP security.

### 1.2. Our Contribution

SSP negotiates the authenticated link key for the pairing Bluetooth devices and employs the NC, OOB, and PE models to prevent MITM attacks. In this paper, the contribution of the SSP security and its HAE applications can be summarized as follows. Firstly, we develop a formal security model to evaluate the security of both the authenticated link key and the NC, OOB, and PE models. Unlike previous work, our security model is applicable to the formal analysis of all SSP protocols. Secondly, we mainly analyze the NC protocol and the OOB protocol under our formal security model and present the security requirements for the basic cryptographic modules in these protocols. Above results will contribute to not only the improvements on the SSP protocols but also the design of their basic cryptographic modules. Thirdly, we explore the typical SSP applications in the HAE systems and propose three principles for SSP, when the security promise is the necessity for the HAE systems. In addition, we compare the related SSP protocols in the aspects of the security and the efficiency and design the SSP implementation plans for the HAE systems.

To our best knowledge, it lacks the formal security model, which can evaluate all SSP protocols under the Bluetooth systems. However, in practice, the highly secure level of the cryptographic protocol must provide the security proof in the formal security model. A substantial contribution of our research is a formal security model for all SSP protocols, which also can be reused to evaluate the similar protocols in future Bluetooth standard. And the security proofs of the NC protocol and the OOB protocol demonstrate that the formal security model can be effectively applied. Although SSP has been used in many HAE systems, no literature gives the tutorial rules about how to deploy SSP. Note that the implementation costs of SSP are expensive for devices. Our principles for SSP aim to fill in gaps and provide the Bluetooth engineers with meaningful guidance.

### 1.3. Notation

The standard notation as in Table 1 is used throughout the rest of the paper. Some notation will be defined locally near its first use, and other notation will be used without further definition.

## 2. Secure Simple Pairing

### 2.1. Overview

SSP consists of five consecutive phases in the following.

Phase 1: Public key exchange
Phase 2: Authentication stage 1
Phase 3: Authentication stage 2
Phase 4: Link key calculation
Phase 5: LMP (link manager protocol) authentication and encryption

The NC protocol and the OOB protocol respectively employ different mechanisms in Phase 2. Nevertheless, other phases are totally the same. And Phase 5 is responsible for authentication and generation of the encryption key. We will omit Phase 5, because Phase 5 is treated as an independent security mechanism and we do not investigate the security of Phase 5.

### 2.2. Numeric Comparison Protocol

The NC protocol Π_1_ is illustrated in Table 2. Any pair of Bluetooth devices A and B can authenticate each other and negotiate a LK after a run of Π_1_.

### 2.3. Out of Band Protocol

Phases 1, 3, and 4 of the OOB protocol Π_2_ are same as those of the NC protocol Π_1_ in Table 2. However, Π_2_‘s Phase 2 uses the OOB mechanism instead of the NC mechanism in Π_1_’s Phase 2. The OOB mechanism in Π_2_ is shown in Table 3.

## 3. Security Model

In the following, we propose a formal security model to evaluate the SSP protocols. Our formal security model generally simulates the networking of the Bluetooth devices and catches the potential attack behaviours when an SSP protocol is run over public channels. Our formal security model can slightly be adjusted to fit any particular deployment model. Of course, it is suitable for the Bluetooth applications in the HAE systems.

### 3.1. Background

We adapt the idea of the secure key exchange definitions [16,17,18,19,20,21] to our setting. Although the basic ideas are similar, there are some of fundamental differences between our model and the classic model for key exchange.

Firstly, the Bluetooth devices do not interact via remote channels, such as Internet channel. Therefore, the users are able to carry out a short numeric comparison, monitor the OOB procedure, or enter the passkey through the Bluetooth devices. This feature can be used to prevent the adversary from carrying out the traditional MITM attack. In the following, we respectively model the numeric comparison and monitoring the OOB procedure as part of the NC protocol and the OOB protocol.

(1) For the NC protocol. Each Bluetooth device participating in a key exchange holds a local public ‘comparison variable’. This variable is public in the sense that the adversary can read its value as his wish. Moreover, the comparison variable can be generated and showed only once in any instance of the NC protocol. The user, therefore, can compare the number and respond ‘yes’ or ‘no’ only once. Obviously, multiple comparisons have more limited use in practice and alert the possible attack.

(2) For the OOB protocol. The adversary is able to only listen to the messages in the OOB channel. The adversary is not allowed to inject, modify, and delete messages over the OOB channel.

Secondly, SSP does not have public-key infrastructure or secret setup information. This is in contrast to the classic shared secret setting, where each pair of parties hold a shared secret key. Despite this, SSP is supposed to be secure in the presence of both active adversary and passive adversary.

Thirdly, the classic model for key exchange focuses on the adversary to learn the secret key that one of the parties obtains at the end of a protocol execution. Comparatively, our model requires the adversary only succeeds if he not only learns the ink key but also passes the authentication procedure of the association model according to Phase 2 in each protocol execution.

In our setting, we assume that each Bluetooth device at most runs one instance of the protocol at the same time. That is, we only allow each Bluetooth device to run many instances sequentially. It makes no sense to allow a single Bluetooth device to run many instances simultaneously. The reason is that each Bluetooth device has only one interface for displaying the comparison variable and the OOB channel such as near field communication (NFC) and therefore always is unable to run two or more instances simultaneously.

### 3.2. Adversary

Assume that the adversary can fully control the Bluetooth network system. During the protocol execution, the adversary has the ability to not only passively eavesdrop the messages exchanged by the Bluetooth device but also actively inject, modify, and delete messages of the Bluetooth device. The adversarial power is modeled by giving the adversary oracle access to the protocol instances that are run by the Bluetooth devices. The oracles provided to the adversary are as follows:

(1) Init(A, B): This call initializes the partnered instances Π^A, B^ and Π^B, A^ for Π. Without loss of generality, we always assume A is the initiating device and B is the non-initiating device in the following discussion. If an instance for the device A or the device B is already run, then the Init oracle does nothing; else the Init oracle starts the instances Π^A, B^ and Π^B, A^ as a new protocol execution.

(2) Send(Π^A, B^, M) or Send(Π^B, A^, M): When this Send oracle is called, it sends the message M to the device A’s Π^A, B^ or the device B’s Π^B, A^. The output of the Send oracle is whatever message Π^A, B^ or Π^B, A^ would send after receiving the message M under the current progress of the Π’s execution. The adversary can carry out MITM attacks on the protocol executions by calling the Send oracles.

(3) Execute(A, B): When the Execute oracle is called, a complete protocol execution between the partnered instances Π^A, B^ and Π^B, A^ is run. The output of the Execute oracle is the protocol transcript, i.e., the complete series of messages exchanged by the protocol execution. This oracle simulates the adversary’s ability to passively eavesdrop on the protocol executions. For a secure protocol, the adversary should learn nothing from such oracle calls.

(4) Reveal(Π^A, B^) or Reveal(Π^B, A^): This call outputs LK that the device A’s Π^A, B^ or the device B’s Π^B, A^ generates at the end of this protocol execution. If the device A’s Π^A, B^ or the device B’s Π^B, A^ does not generate LK, the Reveal oracle outputs a null. This oracle allows the adversary to learn the link keys from previous executions. This Reveal oracle simulates the improper exposure of link keys. To achieve the security, the protocol needs to ensure independence of different link keys generated by different protocol executions.

(5) Test(Π^A, B^) or Test(Π^B, A^): This call is used to define the security of the protocol and does not simulate any real adversarial ability. To answer the query, the Test oracle flips a fair random coin b and returns LK if b = 0 or else a random key with the same bit length of LK if b = 1. Note that this random key is chosen independently of the protocol executions. The adversary is only allowed to query the Test oracle once. We require the adversary to guess the random bit b according to the Π’s executions.

### 3.3. Defining Security

The security definition of the SSP protocols is composed of two basic components: correctness and authenticated link key security. We begin by stating the correctness requirement.

For Π, correctness is viewed as the customary requirement properly given honest Bluetooth devices. If any partnered instances Π^A, B^ and Π^B, A^ communicate without adversarial interference (that is, the adversary never invokes the Send oracle to both Π^A, B^ and Π^B, A^), then Π^A, B^ and Π^B, A^ accept each other at the end of the protocol execution (that is, ac_a_ = ac_b_ = true). Accurately,

**Definition 1** (**Correctness**)**.** *An SSP protocol Π is correct if, given any Bluetooth device A and any Bluetooth device B, any polynomial time protocol experiment executed by partnered instances Π^A, B^ and Π^B, A^ succeeds with overwhelming probability. Here, the polynomial time protocol experiment is successful if and only if ac_a_ = ac_b_ = true at the end of the run of instances Π^A, B^ and Π^B, A^ and the Bluetooth device A and the Bluetooth device B are legitimate*.

It is a challenge to propose a unified security definition for Π, because Π may use NC, OOB, or PE in its Phase 2. Our idea is to divide the security definition into two subparts, i.e., authentication security and link key security. Moreover, although Π perhaps employs different association models in its Phase 2, our security definition merely focuses on the result of authentication processes.

We now define what it means for Π to be secure. Assume that the adversary 𝒜 did not query Reveal(Π^A, B^) or Reveal(Π^B, A^), where the instances Π^A, B^ and Π^B, A^ are partnered. Formally, we say that the adversary 𝒜 succeeds if the following two conditions are all fulfilled:

Condition 1. Both Π^A, B^ and Π^B, A^ finally accept each other, i.e., ac_a_ = ac_b_ = true.

Condition 2. The adversary 𝒜 calls Test(Π^A, B^) or Test(Π^B, A^) and then outputs a bit b_guess_. If the bit b_guess_ is equal to the fair random coin b in the Test oracle, then the adversary 𝒜 succeeds; else the adversary 𝒜 fails.

The adversary 𝒜’s advantage is defined by:

Adv(𝒜) = |2Prob(𝒜 succeeds in achieving both Condition 1 and Condition 2) − 1|.(1)

Explanation. Intuitively, an SSP protocol is secure if the adversary 𝒜 cannot distinguish real link keys from random ones. This means that the Bluetooth devices can securely use their link keys to establish secure channels. For more discussions on this issue, we refer to see [16,17,18]. Due to the SSP protocols, we demand that the adversary 𝒜 correctly guesses the fair random coin b in either the Test(Π^A, B^) oracle or the Test(Π^B, A^) oracle, if and only if both Π^A, B^ and Π^B, A^ accept each other. The reason is that the Bluetooth user will reject the paring result and at least one of Π^A, B^ and Π^B, A^ does not output the link key if Π^A, B^ or Π^B, A^ unsuccessfully finishes. Moreover, the user should find this abnormal case and terminate the subsequent Bluetooth communication. In addition, the adversary 𝒜 can always correctly guess the fair random coin b in a Test(Π^A, B^) or Test(Π^B, A^) query if he queried Reveal(Π^A, B^) or Reveal(Π^B, A^) before. This is a trivial case. Hence, the adversary 𝒜 is only said to have succeeded if these Reveal oracles were not queried.

Let n or 1^n^ be the security parameter. A real-valued function f: N → [0, 1] is negligible if for every polynomial p() there exists an integer N such that for every n > N it holds that f(n) < 1/p(n). In the following, we denote an arbitrary negligible function by negl. We define the notion of authenticated link key security as follows.

**Definition 2** (**Authenticated link key security**)**.** *For an SSP protocol Π, the adversary 𝒜 starts with a learning phase allowing the polynomial time queries of the Init, Send, Execute, and Reveal oracles for any pairing Bluetooth device. The adversary 𝒜 then enters into an attack phase pursuing with a Test oracle query. The adversary 𝒜 provides the partnered instances Π^A, B^ and Π^B, A^ related to Bluetooth device A and Bluetooth device B, where the Reveal oracle is not queried to the instances Π^A, B^ and Π^B, A^. Now, the adversary 𝒜 calls Test(Π^A, B^) or Test(Π^B, A^) and then outputs a bit b_guess_. The SSP protocol Π is secure for authenticated link key if there exists a negligible function negl such that*

*Prob(𝒜 succeeds) = Prob(𝒜 submits Π^A, B^ and Π^B, A^ and his bit b_guess_ such that ac_a_ = ac_b_ = true and b_guess_ = b)* < ½ *+ negl(n)*, (2)

*where ac_a_ and ac_b_ are the decisions in the partnered instances Π^A, B^ and Π^B, A^, b is the fair random coin in the Test oracle, and n is the security parameter of Π.*

Definition 2 does not show an obvious way to prove the SSP protocols secure. Our trick of the proof is to classify the active adversary and the passive adversary and respectively prove the intermediate security results of the SSP protocols under the active adversary and the passive adversary. We can use these intermediate security results to deduce the final security results of the SSP protocols, which satisfy Definition 2. The detailed analysis is in the following.

In the learning phase of Definition 2, it is clear that the Send, Init, and Reveal oracles queries are active behavior, however, the Execute oracle queries are passive behavior. Hence, the adversary *𝒜* further can be divided into the active adversary 𝒜_1_ and the passive adversary 𝒜_2_ as follows.

(1) Active adversary 𝒜_1_: As Definition 2, 𝒜_1_ must send at least one fabricated message to Π^A, B^ or Π^B, A^. Here, 𝒜_1_ can use the Init and Send oracles to realize it during the learning phase.

(2) Passive adversary 𝒜_2_: As Definition 2, 𝒜_2_ never sends any his own fabricated message to both Π^A, B^ and Π^B, A^ during the learning phase. In fact, 𝒜_2_ functions just like a wire, even he can invoke the Init and Send oracles.

**Theorem** **1.** *Consider an SSP protocol Π under Definition 2. Let E_1_ denote the event that the active adversary 𝒜_1_ submits the partnered instances Π^A, B^ and Π^B, A^ such that ac_a_ = ac_b_ = true. Let E_2_ denote the event that the passive adversary 𝒜_2_ submits the partnered instances Π^A, B^ and Π^B, A^, calls Test(Π^A, B^) or Test(Π^B, A^), and outputs the bit b_guess_ such that b_guess_ = b. We have*

*Prob(𝒜 succeeds) = Prob(𝒜 submits Π^A, B^ and Π^B, A^ and his bit b_guess_ such that ac_a_ = ac_b_ = true and b_guess_ = b) ≤ Prob(E_1_) + Prob(E_2_).* (3)

**Proof** **1.** According to Definition 2, it holds that

Prob(𝒜 submits Π^A, B^ and Π^B, A^ and his bit b_guess_ such that ac_a_ = ac_b_ = true and b_guess_ = b) ≤ Prob(𝒜_1_ achieves ac_a_ = ac_b_ = true and b_guess_ = b for Π^A, B^ and Π^B, A^) + Prob(𝒜_2_ achieves ac_a_ = ac_b_ = true and b_guess_ = b for Π^A, B^ and Π^B, A^). (4)

Since the passive adversary 𝒜_2_ never sends any his own fabricated message to both Π^A, B^ and Π^B, A^ in the learning phase, both ac_a_ and ac_b_ in Π^A, B^ and Π^B, A^ are always true at the end of the protocol execution. It further means that

Prob(𝒜 submits Π^A, B^ and Π^B, A^ and his bit b_guess_ such that ac_a_ = ac_b_ = true and b_guess_ = b) ≤ Prob(𝒜_1_ achieves ac_a_ = ac_b_ = true for Π^A, B^ and Π^B, A^) + Prob(𝒜_2_ achieves b_guess_ = b for Π^A, B^ and Π^B, A^). (5)

Note that we do not demand the active adversary *𝒜*_1_ guessing the fair random coin b in the Test oracle. We now obtain the Equation (3) and complete the proof of theorem. □

Theorem 1 tells us that the analysis of authenticated link key security can be simplified as two independent games, i.e., G_1_ and G_2_. We explain them as follows. The game G_1_ under any Π plays with the active adversary 𝒜_1_, who uses the Init, Send, Execute, and Reveal oracles and tries to modify the message(s) between any partnered instances of Π during the learning phase. In the attack phase of Definition 2, the goal of the active adversary 𝒜_1_ is to provide his fabricated instances Π^A, B^ and Π^B, A^, which successfully pass the authentication procedure of Phase 2 and Phase 3 in Π. Comparatively, the game G_2_ in the learning phase of Definition 2 interacts with the passive adversary 𝒜_2_, who never modifies the messages between any partnered instances of Π. The goal of the passive adversary 𝒜_2_ is to distinguish the link key of a partnered instances Π^A, B^ and Π^B, A^ from a random key.

## 4. Correctness of Results

### 4.1. Correctness

**Theorem** **2.** *As described in Table 2 and Table 3, Π_1_ and Π_2_ are all correct under Definition 1.*

**Proof** **2.** Assume that any Bluetooth device A and any Bluetooth device B under Π_1_ are legitimate and the adversary does not interfere the Bluetooth devices’ Π_1_^A, B^ and Π_1_^B, A^. In Π_1_’s Phase 2, Π_1_^A, B^ should successfully check Cb = f1(PKbx, PKax, Nb, 0), and then the user should confirm ‘ok’ due to Va = Vb. Then, in Π_1_’s Phase 3, Π_1_^A, B^ and Π_1_^B, A^ respectively set ac_a_ = true and ac_b_ = true because Π_1_^B, A^ confirms Ea = f3(DHKey, Na, Nb, rb, IOcapA, A, B) and Π_1_^A, B^ confirms Eb = f3(DHKey, Nb, Na, ra, IOcapB, B, A). Therefore, Π_1_ is correct according to Definition 1. For Π_2_, we can get a similar result for any partnered Π_2_^A, B^ and Π_2_^B, A^, i.e., ac_a_ = true and ac_b_ = true. Those complete the proof. □

### 4.2. Authenticated Link Key Security

Assume that the Diffie-Hellman key over the group G is generated by the Diffie-Hellman key exchange. The well-known Decisional Diffie-Hellman (DDH) assumption [40] states that the Diffie-Hellman key is indistinguishable from a random element in the group G. The DDH assumption is formally defined as follows.

**Definition** **3.** *Let gen(1^n^) be a parameter generation algorithm that outputs the description of a group G, its generator P ∈ G, and its order q. We say the DDH problem is hard relative to G if for all probabilistic polynomial time algorithms D there exists a negligible function negl such that*

*|Prob(D(gen(1^n^), a⋅P, b⋅P, ab⋅P) = 1) − Prob(D(gen(1^n^), a⋅P, b⋅P, c⋅P) = 1)| < negl(n)*, (6)

*where a, b, and c are randomly chosen in {1, … , q}.*

We say that a keyed function H_1_(k, ) is the Diffie-Hellman keyed pseudorandom function if H_1_(k, ) is a keyed pseudorandom function when its key k is a Diffie-Hellman key in a certain group G. We formally write it in Definition 4.

**Definition** **4.** Let gen(1^n^) be a parameter generation algorithm that outputs the description of a group G, its generator P ∈ G, and its order q. Let H_1_(k, ) be a keyed pseudorandom function using Diffie-Hellman key if for every probabilistic polynomial time distinguisher D* there exists a negligible function negl such that

|Prob(D*(gen(1^n^), a⋅P, b⋅P, H_1_(ab⋅P, )) = 1) − Prob(D*(gen(1^n^), a⋅P, b⋅P, R()) = 1)| < negl(n), (7)

*where a and b are randomly chosen in {1, … , q} and R is a truly random function.*

Note that a rigorous pseudorandom function receives a uniformly distributed input. Therefore, it does not necessarily suffice for Definition 4, that is, a random element of G is not necessarily a uniformly distributed bit string. We also need two standard assumptions [40] for the cryptographic hash functions as follows.

**Definition** **5.** *Let gen(1^n^) be a parameter generation algorithm that outputs the description of a group G, its generator P ∈ G, and its order q. Let a and b be randomly chosen in {1, … , q}. Let H_2_(ab⋅P, ) be a message authentication code (MAC) function using Diffie-Hellman key if for every probabilistic polynomial time algorithm A there exists a negligible function negl such that*

*Prob(A(H_2_(ab⋅P, )) outputs (x, h) such that h = H_2_(ab⋅P, x) and x ∉ Q) < negl(n)*, (8)

*where Q denotes all H_2_(ab⋅P, ) oracle queries asked by A and n is the security parameter of H_2_(ab⋅P, ).*

**Definition** **6.** Let H_3_() be a collision-resistant function if for every probabilistic polynomial time algorithm A* there exists a negligible function negl such that

Prob(A*(H_3_()) outputs (x_0_, x_1_) such that H_3_(x_0_) = H_3_(x_1_)) < negl(n), (9)

*where n is the security parameter of H_3_().*

To reduce our security results, we firstly require proving three security facts for Π, Π_1_, and Π_2_ as follows.

**Lemma** **1.** *Assume that P256() and P192() in Π satisfy the DDH assumption as in Definition 3 and f2() and f3() in Π are the Diffie-Hellman keyed pseudorandom function as in Definition 4. Let 𝒜_2_ be the passive adversary as described in Section 3.3. Then, Π_1_ and Π_2_ under the passive adversary 𝒜_2_ are all secure according to Definition 2.*

**Proof** **3.** We know that the passive adversary 𝒜_2_ during the learning phase in Definition 2 does not send his own fabricated message to both Π^A, B^ and Π^B, A^. Therefore, the passive adversary 𝒜_2_ can collect the transcripts of the protocol executions including those of Π^A, B^ and Π^B, A^. And then, to violate the security of Π, the passive adversary 𝒜_2_ during the attack phase in Definition 2 must strictly depend on correctly guessing the fair random coin b in the Test(Π^A, B^) or Test(Π^B, A^) oracle. That is, for any passive adversary 𝒜_2_ interacting with the Bluetooth device A and the Bluetooth device B, the secure Π should satisfy that

Prob(𝒜_2_ succeeds) = Prob(𝒜_2_ submits Π^A, B^ and Π^B, A^ and his bit b_guess_ such that b_guess_ = b) < ½ + negl(n). (10)

Recall that Π_1_ and Π_2_ have totally same Phases 1, 3, and 4. The only difference is that Π_1_ uses the NC mechanism in Phase 2 and Π_2_ uses the OOB mechanism in Phase 2. However, the passive adversary 𝒜_2_ does not exploit the weaknesses of these mechanisms, because they have no use of the Diffie-Hellman key. Moreover, both Π_1_‘s Phase 2 and Π_2_‘s Phase 2 finally output the random numbers Na and Nb as the inputs of their subsequent phases. Therefore, in the view of the passive adversary 𝒜_2_, Π_1_ and Π_2_ are same. Based on this observation, we can conformably present the formal proof for Π_1_ and Π_2_. The formal reduction for them is as follows.

Let 𝒜_2_ be a probabilistic polynomial time passive adversary and let δ_1_ be a function such that

Prob(𝒜_2_ succeeds) = Prob(𝒜_2_ submits Π^A, B^ and Π^B, A^ and his bit b_guess_ such that b_guess_ = b) < ½ + δ_1_(n). (11)

We demonstrate that δ_1_ is negligible by presenting a DDH problem distinguisher D_1_ with the same advantage δ_1_. The distinguisher D_1_ receives (a⋅P, b⋅P, K) and attempts to determine if K = ab⋅P or K is a random element in the group G. The distinguisher D_1_ simulates Π’s Π^A, B^ and Π^B, A^ in the following:

Π’s Phase 1. When the passive adversary 𝒜_2_ calls the Send oracle to the Bluetooth device A for the public key, the distinguisher D_1_ sends PKa = a⋅P to him. When the passive adversary 𝒜_2_ calls the Send oracle to the Bluetooth device B for the public key, the distinguisher D_1_ sends PKb = b⋅P to him. Here, the passive adversary 𝒜_2_ should honestly resend PKa to the Bluetooth device B and PKb to the Bluetooth device A, because the passive adversary 𝒜_2_ does not modify any message.

Π’s Phase 2. The distinguisher D_1_ acts exactly like the Bluetooth device A and the Bluetooth device B would.

Π’s Phase 3. The distinguisher D_1_ uses its K as the Diffie-Hellman key. That is, the distinguisher D_1_ computes Ea = f3(K, Na, Nb, rb, IOcapA, A, B) and Eb = f3(K, Nb, Na, ra, IOcapB, B, A) and sends them.

Π’s Phase 4. Like Π’s Phase 3, the distinguisher D_1_ computes LK by f2(K, Na, Nb, ”btlk”, BD_ADDRa, BD_ADDRb).

When the passive adversary 𝒜_2_ queries Test(Π^A, B^) or Test(Π^B, A^), the distinguisher D_1_ chooses a random bit b and replies with LK if b = 0 and with a random number with the same bit-length of LK if b = 1. Finally, the distinguisher D_1_ outputs 1 when the passive adversary 𝒜_2_ outputs b_guess_ = b.

In the view of the passive adversary 𝒜_2_, above simulation by the distinguisher D_1_ is exactly a Π’s execution, when K = ab⋅P. Hence, we know

Prob(D_1_(gen(1^n^), a⋅P, b⋅P, ab⋅P) = 1) = ½ + δ_1_(n). (12)

When K is a random element in the group G, the parameters Ea, Eb, and LK are calculated using the random element K instead of ab⋅P. Let δ_2_(n) be a function such that the passive adversary 𝒜_2_ outputs b_guess_ = b with probability 1/2 + δ_2_(n), where δ_2_(n) is the advantage by exploiting Ea, Eb, and LK. We show that δ_2_ is a negligible function and argue it by contradiction. Assume that δ_2_ is a non-negligible function. We can construct the distinguisher D_2_ to tell keyed pseudorandom function using Diffie-Hellman key from truly random function. The distinguisher D_2_ generates its own (SKa, PKa), (SKb, PKb), and DHKey and simulates the Π’s execution. In the distinguisher D_2_’s simulation, the keyed pseudorandom functions f3(DHKey, ) and f2(DHKey, ) or the truly random functions are used to compute Ea, Eb, and LK. If the truly random functions exist, the passive adversary 𝒜_2_ outputs correct guess with probability 1/2. If the keyed pseudorandom functions f3(DHKey, ) and f2(DHKey, ) are given to the distinguisher D_2_, it is the same as the case of the distinguisher D_1_ with the random K. Hence, in this case, the distinguisher D_2_ outputs correct guess with probability 1/2 + δ_2_(n). This implies that the advantage of the distinguisher D_2_ is a non-negligible function δ_2_(n), contradicting Definition 4. Hence, δ_2_(n) must be a negligible function. Now, we further have

|Prob(D_1_(gen(1^n^), a⋅P, b⋅P, ab⋅P) = 1) − Prob(D_1_(gen(1^n^), a⋅P, b⋅P, K) = 1)| = |½ + δ_1_(n) − ½ − δ_2_(n)| = | δ_1_(n) − δ_2_(n)|.      (13)

According to Definition 3, we know that δ_1_(n) is also a negligible function due to the negligible function δ_2_(n). Thus, we conclude that Π_1_ and Π_2_ under the passive adversary 𝒜_2_ are all secure for authenticated link key. □

**Lemma** **2.** *Assume that as defined in [25], f1() is the computationally-binding non-malleable commitment scheme and g() is a computational 2-universal hash function. Let f3() be a MAC function as in Definition 5. Let 𝒜_1_ be the active adversary of Π_1_ as described in Section 3.3. Let E_3_ denote the event that 𝒜_1_ submits his Π_1_^A, B^ and Π_1_^B, A^ such that ac_a_ = ac_b_ = true. Then, Prob(E_3_) is negligible*.

**Proof** **4.** Using the Send oracle, the active adversary 𝒜_1_ perhaps generates his own fabricated PK’a, PK’b, N’a, N’b, E’a, and E’b and sends them instead of PKa, PKb, Na, Nb, Ea, and Eb to the corresponding Π_1_^A, B^ and Π_1_^B, A^. Two cases need be considered as follows.

Case 1. The active adversary 𝒜_1_ alters the message(s) in Π_1_’s Phase 1. Let the output of the function g be l-bit length. Let E_31_ denote the event that g(PKa, PK’b, Na, N’b) = g(PK’a, PKb, N’a, Nb) and either PKa ≠ PK’a or PKb ≠ PK’b or both, where the active adversary 𝒜_1_’s PK’a, PK’b, N’a, and N’b are sent by calling the Send oracle(s) during the run of Π_1_^A, B^ and Π_1_^B, A^. In [25], Theorem 4.1 proves that Prob(E_31_) = 2^−l^ + δ_3_(n), where δ_3_ is a negligible function.

Case 2. The active adversary 𝒜_1_ only alters the message(s) in Π_1_’s Phase 2 or (and) Phase 3. Let E_32_ denote the event that the active adversary 𝒜_1_ sends N’a ≠ Na or N’b ≠ Nb or both to the corresponding Π_1_^A, B^ and Π_1_^B, A^ and ac_a_ = ac_b_ = true after Π_1_’s Phase 3. Let δ_4_ be a function such that Prob(E_32_) = δ_4_(n). We demonstrate that the function δ_4_ is negligible by presenting a forged MAC generator GT with the same advantage δ_4_. The forged MAC generator GT receives the description of the MAC function f3(DHKey, ) and further attempts to at least generate new Mac pair (x, h) such that h = f3(DHKey, x). The forged MAC generator GT simulates Π_1_^A, B^ and Π_1_^B, A^ as follows.

1. The forged MAC generator GT performs the same operations as the Bluetooth device A and the Bluetooth device B in Π_1_’s Phase 1 and Phase 2. That is, the forged MAC generator GT randomly chooses (SKa, PKa) and (SKb, PKb), sends PKa and PKb, and computes DHKey in Π_1_’s Phase 1. And then, the forged MAC generator GT randomly selects Na and Nb and sends Cb, Na, and Nb in Π_1_’s Phase 2.

2. The forged MAC generator GT observes that the active adversary 𝒜_1_ sends his own N’a or (and) N’b instead of Na or (and) Nb to the corresponding Π_1_^B, A^ or (and) Π_1_^A, B^. Here, 𝒜_1_ can implement it by calling Send(Π_1_^B, A^, N’a) or (and) Send(Π_1_^A, B^, N’b).

3. In Π_1_’s Phase 3, the forged MAC generator GT computes and sends Ea = f3(DHKey, Na, N’b, rb, IOcapA, A, B) or (and) Eb = f3(DHKey, Nb, N’a, ra, IOcapB, B, A). The forged MAC generator GT observes that the active adversary 𝒜_1_ sends his own E’a or (and) E’b instead of Ea or (and) Eb. And then, based on receiving N’a or (and) N’b, the forged MAC generator GT checks whether E’a = f3(DHKey, N’a, Nb, rb, IOcapA, A, B) or (and) E’b = f3(DHKey, N’b, Na, ra, IOcapB, B, A). If all are correct, the forged MAC generator GT sets ac_b_ = true and ac_a_ = true; else it sets at least one of ac_b_ and ac_a_ is false.

4. In Π_1_’s Phase 4, the forged MAC generator GT acts as the Bluetooth device A and the Bluetooth device B.

In the view of the active adversary 𝒜_1_, the forged MAC generator GT constructs a perfect simulation of the run of Π_1_^A, B^ and Π_1_^B, A^. When ac_a_ = ac_b_ = true, the forged MAC generator GT obtains at least one new MAC pair without using f3(DHKey, ), i.e., ({N’a, Nb, rb, IOcapA, A, B}, E’a) or ({N’b, Na, ra, IOcapB, B, A}, E’b). Because f3(DHKey, ) is a MAC function, δ_4_ is a negligible function due to Definition 5.

We know that the event E_3_ needs that the message(s) of Π_1_^A, B^ and Π_1_^B, A^ must be modified by the active adversary 𝒜_1_ and at the same time Π_1_^A, B^ and Π_1_^B, A^ still have ac_a_ = ac_b_ = true after Π_1_’s Phase 3. Nevertheless, 𝒜_1_ in the event E_31_ needs to change either PKa or PKb or both in the corresponding Π_1_^B, A^ and Π_1_^A, B^ to satisfy the comparison procedure of Π_1_’s Phase 2 and 𝒜_1_ in the event E_32_ changes the message(s) of Π_1_^A, B^ and Π_1_^B, A^ except PKa and PKb. Hence, we have

Prob(E_3_) *≤* Prob(E_31_) + Prob(E_32_) = 2^−l^ + δ_3_(n) + δ_4_(n). (14)

It implies that Prob(E_3_) is negligible in the security parameters l and n. □

**Lemma** **3.** *Assume that f1() in Π_2_ is the collision-resistant function as in Definition 6. Assume that f3() in Π_2_ is the MAC function as in Definition 5. Let 𝒜_1_ be the active adversary of Π_2_ as described in Section 3.3. Assume that 𝒜_1_ is not allowed to modify Π_2_‘s messages {A, ra, Ca} and {B, rb, Cb} transmitted over the OOB channels. Let E_4_ denote the event that the active adversary 𝒜_1_ submits his Π_2_^A, B^ and Π_2_^B, A^ such that ac_a_ = ac_b_ = true. Then, Prob(E_4_) is negligible.*

**Proof** **5.** The active adversary 𝒜_1_ should fabricate at least one message to his submitting Π_2_^A, B^ and Π_2_^B, A^ and the decisions of both Π_2_^A, B^ and Π_2_^B, A^ still satisfy ac_a_ = ac_b_ = true. Let us analyze the behaviour of the active adversary 𝒜_1_. For Π_2_^A, B^ and Π_2_^B, A^, 𝒜_1_ may modify the message(s) transmitted in Π_2_’s Phase 1 or Phase 2. Note that if the active adversary 𝒜_1_ wants to modify Π_2_’s messages in both Phase 1 and Phase 2, he should firstly pass the authentication procedure in Π_2_’s Phase 2. We therefore do not consider this case. Now, we need to consider two cases as follows.

Case 1. In Π_2_’s Phase 1, the active adversary 𝒜_1_ modifies Π_2_^A, B^’s PKa or (and) Π_2_^B, A^’s PKb. Let the event E_41_ denote that the active adversary 𝒜_1_ only uses the Send oracle to transmit his PK’a = (PK’ax, PK’ay) instead of PKa to Π_2_^B, A^ and Π_2_^B, A^‘s ac_b_ is not false, where PK’a ≠ PKa. We know that Prob(𝒜_1_ modifies message(s) of Π_2_^A, B^ or (and) Π_2_^B, A^ in Phase 1) ≤ Prob(E_41_) according to the design of Π_2_. In Step 5 of Π_2_’s Phase 2, the Bluetooth device B receives ra and Ca from the Bluetooth device A and sets ac_b_ = false and aborts the protocol execution if Ca ≠ f1(PK’ax, PK’ax, ra, 0). It means that, if the event E_41_ holds, we obtain two different inputs {PK’ax, PK’ax, ra, 0} and {PKax, PKax, ra, 0} for the collision-resistant function f1 such that Ca = f1(PKax, PKax, ra, 0) = f1(PK’ax, PK’ax, ra, 0). Due to Definition 6, we have Prob(E_41_) = δ_5_(n), where δ_5_(n) is a negligible function.

Case 2. For Π_2_^A, B^ or (and) Π_2_^B, A^, the active adversary 𝒜_1_ only modifies the message(s) in Π_2_’s Phase 2 and Phase 3. Let E_42_ denote the event that the active adversary 𝒜_1_ sends N’a ≠ Na or N’b ≠ Nb or both to the corresponding Π_2_^B, A^ and Π_2_^A, B^ and both ac_b_ and ac_a_ are true after Π_2_’s Phase 3. Clearly, Prob(𝒜_1_ only modifies message(s) of Π_2_^A, B^ or (and) Π_2_^B, A^ in Phase 2 and Phase 3) ≤ Prob(E_42_). Let δ_6_ be a function such that Prob(E_42_) = δ_6_(n). Using a similar trick in case 2 of Lemma 2, we reduce that δ_6_ is a negligible function by using Definition 5.

To conclude the proof, we have

Prob(E_4_) ≤ Prob(𝒜_1_ modifies message(s) of Π_2_^A, B^ or (and) Π_2_^B, A^ in Phase 1) + Prob(𝒜_1_ only modifies message(s) of Π_2_^A, B^ or (and) Π_2_^B, A^ in Phase 2 and Phase 3) ≤ Prob(E_41_) + Prob(E_42_) = δ_5_(n) + δ_6_(n), (15)

where both δ_5_(n) and δ_6_(n) are negligible functions. This completes the proof. □

Combining Theorem 1 with Lemma 1, Lemma 2, and Lemma 3, we can directly obtain the following results of Π_1_ and Π_2_ under our security model.

**Theorem** **3.** *Assume that P256() and P192() in Π satisfy the DDH assumption as in Definition 3 and f2() and f3() in Π are the Diffie-Hellman keyed pseudorandom function as in Definition 4. We have*

*(1)*  *Π_1_ is secure for authenticated link key, if f1() is the computationally-binding non-malleable commitment scheme, g() is a computational 2-universal hash function, and f3() is also a MAC function as in Definition 5*.
*(2)*  *Π_2_ is secure for authenticated link key, if f1() is the collision-resistant function as in Definition 6 and f3() is also a MAC function as in Definition 5.*

## 5. Comparison Analysis of Related Protocols

In this section, we analyze the security properties and the efficiency of the NC protocol and the OOB protocol in Bluetooth standard v5.0 by contrast to three related SSP protocols [12,13,23].

### 5.1. Security Properties Comparison

The improved NC protocol proposed by Yeh et al. [23] uses PIN number instead of confirming the displayed numbers to prevent MITM attacks. The improved NC protocol proposed by Gajbhiye et al. [12] uses the signature mechanism to confirm the pairing devices. The SSP protocol with delayed-encrypted IO (SSP-DEIO) proposed by Gajbhiye et al. [13] delays the exchange of encrypted IO capability until Phase 2 to overcome from the problem of capturing the IO capability of the pairing devices. Table 4 shows the security comparison among our results and above three SSP protocols. Here, ‘Yes’/‘No’ represents that the protocol can/cannot defeat the corresponding attack. As shown in Table 4, our NC and OOB protocols show better security properties against passive eavesdropping and MITM attacks. More important, these properties have been strictly proved by the formal security model.

### 5.2. Performance Comparison

We compare and implement all the SSP protocols under the same experiment platform. We know that the NC protocol requires the user to carry out a short numeric comparison and the OOB protocol needs the user to monitor the OOB procedure. Due to the uncertainty of human factors, we omit the performance of these user-device interactions in all the SSP protocols.

#### 5.2.1. Computation Cost

##### Experiment Platform

(1) Experiment environment: The basic cryptographic algorithms are executed in Windows 10 64 bits, Intel(R) Core(TM) i7-8700 CPU @ 3.20 GHz 3.19GHz, 8.00 GB RAM.

(2) Cryptographic tools: Python 3.6.6 cryptography toolkit PyCryptodemo.

##### Parameters and Algorithms

Several parameters are involved in the different SSP protocols. Based on the security assumption of our formal security model in Section 4 and Bluetooth standard v5.0, these parameters and their corresponding algorithms are shown in Table 5.

**Figure 1:**
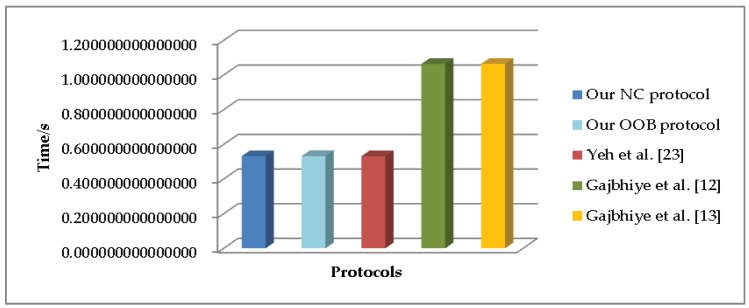
Comparison of computation time cost.

Table 6 shows the total computation operations of five SSP protocols. Figure 1 depicts the differences of the computation time cost between these SSP protocols.

#### 5.2.2. Communication and Storage Cost

**Figure 2:**
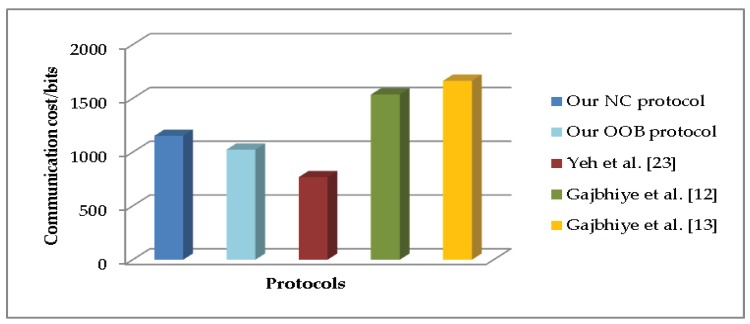
Comparison of communication cost.

**Figure 3:**
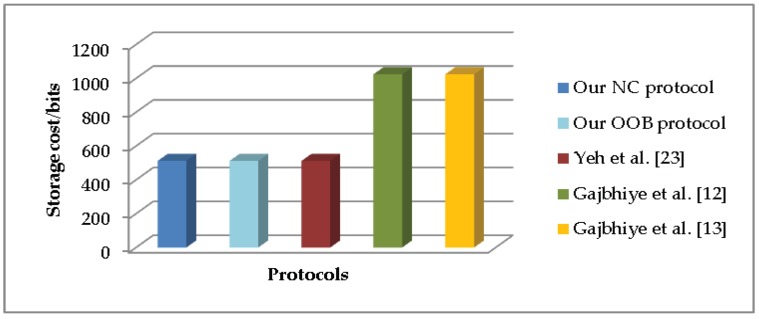
Comparison of storage cost.

To evaluate the communication cost, we consider the transmitted data in each protocol execution. Figure 2 shows the communication cost of all the SSP protocols. In the aspect of the storage cost, we only calculate the long-standing value existed in all phases of the SSP protocols. Figure 3 compares the storage cost of all the SSP protocols.

## 6. Security Applications in Home Automation and Entertainment

**Figure 4:**
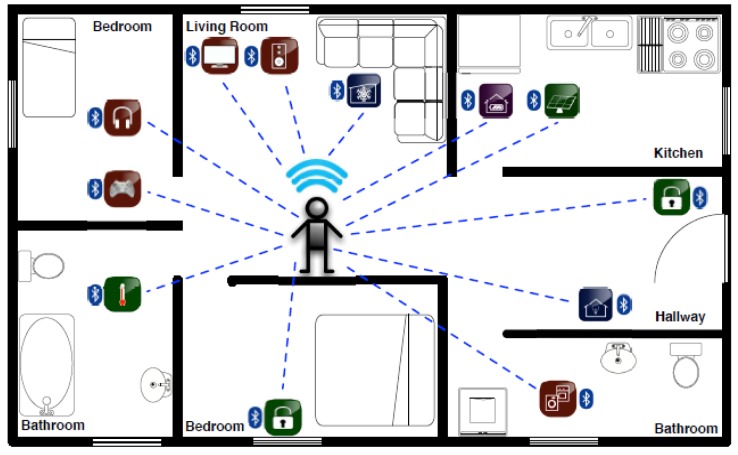
Bluetooth-based home automation and entertainment (HAE) system.

Bluetooth has been widely used by the HAE systems due to its flexibility of networking and diversity of connection. Figure 4 shows a typical Bluetooth-based HAE system. On the one hand, users via Bluetooth enable automatic control of multiple household items, including lights, washing machines, thermostats, smoke detectors, cameras, door bells, locks, and more. On the other hand, Bluetooth connects various home entertainment devices such as TVs, media players, gaming consoles, and virtual reality wearables, and makes smart entertainment a reality. However, the HAE systems are susceptible to all kinds of attacks such as traffic eavesdropping, MITM attacks, and session hijacking. Bluetooth specially maintains the SSP solution to protect its service. Clearly, the SSP solution is possible to prevent the attacks existed in the HAE systems, when these systems are built on the Bluetooth technology.

### 6.1. Integration Frame

To secure the Bluetooth-based HAE systems, the SSP solution must perfectly be integrated with the HAE systems for the strong cryptographic security. We therefore propose an SSP frame for the HAE scenarios. The SSP frame suggests how to correctly apply SSP in the HAE systems. The SSP frame is presented as follows.

**Principle** **1.** *SSP is the basic security component when networking the HAE systems*.

**Figure 5:**
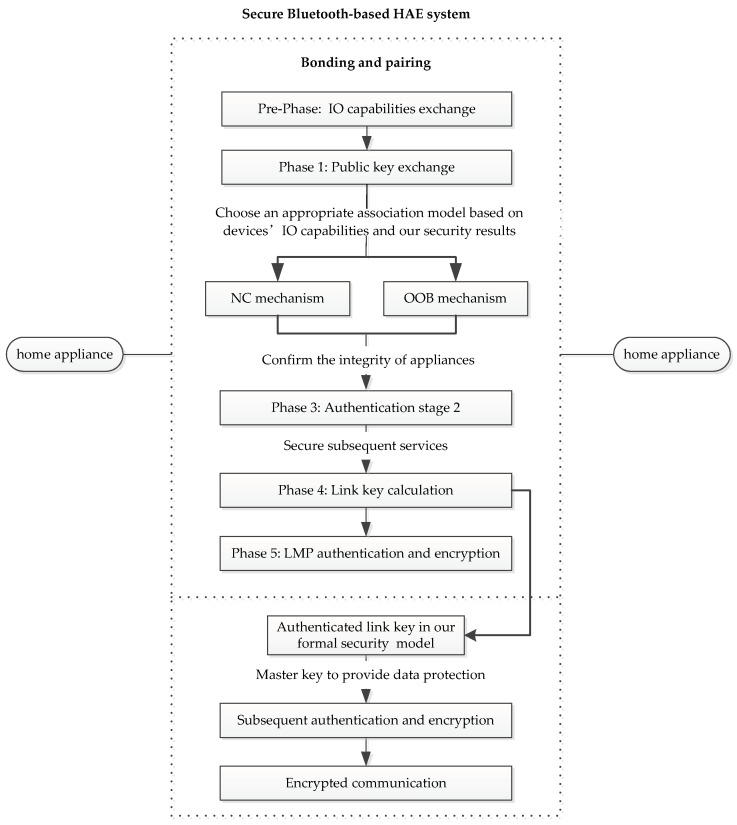
A secure Bluetooth-based HAE system using secure simple pairing (SSP).

Before sensitive communication, the security link should be established among the home appliances in the HAE systems. This security link requires using the crucial pairing and bonding protocol in the SSP solution. As shown in Figure 5, if two appliances in the HAE system need to establish a security link in actual system implementation, they need select SSP as the secure pairing solution. In more detail, both appliances generate their own public-private key pairs and exchange necessary messages. Then, users can choose appropriate association model of SSP to pair their appliances based on their IO capabilities and our security results in Section 4. For example, if the OOB data are available on each pairing appliance and a strong security for authenticated link key is needed, users could select the OOB protocol to pair their home appliances. Users then use Phase 3 in Figure 5 to confirm the integrity of appliances. Here, to secure subsequent services, the link key is perhaps established by the SSP protocol. The link key would be used as a master key to complete sequent authentication and encryption.

**Principle** **2.** *SSP can be used to prevent unauthorized users in the HAE systems, when the unauthorized user is a threat.*

The HAE systems always demand that all home appliances are authorized. It implies that the HAE systems must prevent MITM attacks from malicious appliances. Under our formal evaluation, the NC protocol and the OOB protocol in SSP prevent MITM attacks. More important, to defeat MITM attacks, users only need to perform the simple operations such as comparing two numbers and monitoring the OOB procedure. Based on Theorem 3, the probability that adversary succeeds in carrying out a MITM attack should be negligible.

**Principle** **3.** *SSP can be used to provide the data protection for the HAE systems*.

Many home appliances in the HAE systems process a large amount of private sensitive data. The link keys generated by the SSP protocols are advised to encrypt and authenticate those data. This suggestion is supported by our formal proof of the SSP protocol. Specifically, the link key generated in Phase 4 of the SSP protocol can be used as a master key for sequent authentication and encryption. Owing to Theorem 3, the NC and OOB protocols have been formally proved to satisfy the authenticated link key security. Hence, the secret key can be securely derived by the link key. If the private sensitive data are encrypted by the secret key, the adversary is uneasy to obtain the private sensitive data in the HAE system.

### 6.2. Case Research

#### 6.2.1. Smart Lock

**Figure 6:**
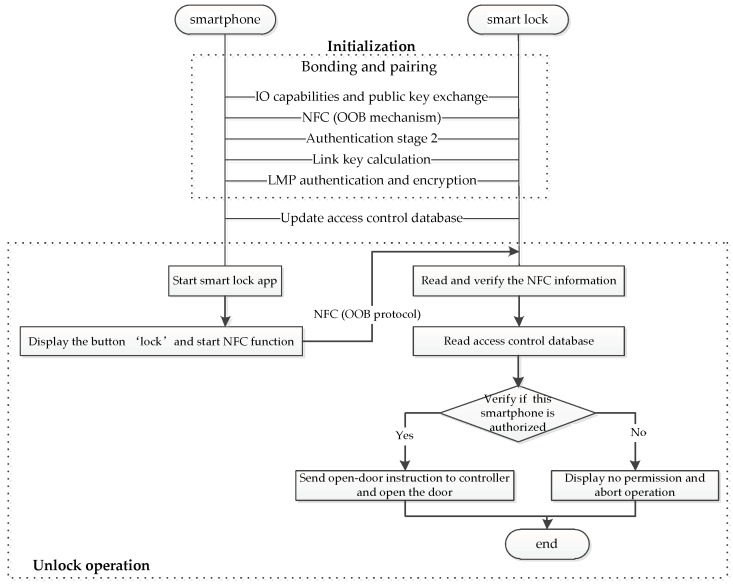
Implementation design of a smart lock system using near field communication (NFC).

The smart lock system is implemented in houses, lockers, and boxes for postal applications, etc. Users’ mobile devices connect with the physical locks via Bluetooth wireless communication. This system helps people to lock and unlock the door automatically, particularly the disabled and elderly people. People can not only control the state of lock but also monitor the malicious intruder using his automation system. Since the smart lock system is an important line of family security, it must ensure that only authorized user has access to the door. The smart lock need adopt Principle 1 and Principle 2. Principle 1 assures users that their mobile devices are able to setup the security links with the target locks. Principle 2 prevents the malicious intruder, who may exploit the appliances in the system. Figure 6 shows the implementation design of smart lock under the OOB protocol. The authorized users pair and bond a smartphone with the smart lock by the aid of the OOB protocol. When a user wants to unlock the smart lock, he should start the smart lock app in his smartphone and start the NFC function to transfer NFC information to the smart lock. Smart lock reads the information and verifies to determine if open the door. Here, NFC plays a vital role in the smart lock system.

#### 6.2.2. Sport and Fitness Wearables

**Figure 7:**
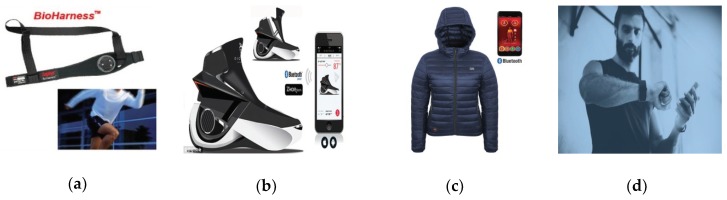
Four sport and fitness wearables: (**a**) Bioharness; (**b**) Smart training shoes; (**c**) Heated jacket; (**d**) Sport monitor.

**Figure 8:**
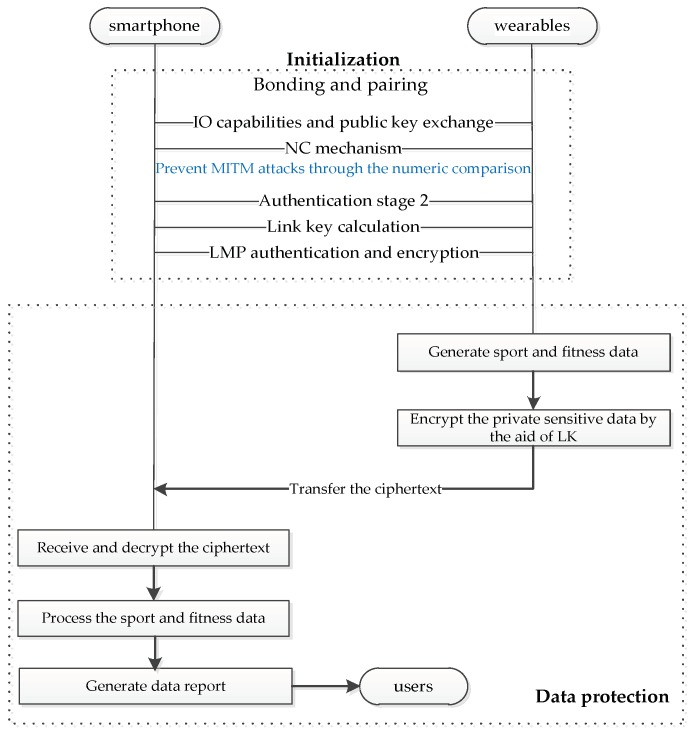
Smart wearables implementation using NC protocol.

Figure 7 shows four sport and fitness wearables, namely bioharness, smart training shoes, heated jacket, and sport monitor. The personal sensitive data are usually stored on wearables. The data protection is important, because the data on wearables are apt to be tracked and eavesdropped by an adversary. Fortunately, users easily pair their sport and fitness wearables with smartphones by easy SSP operations. Hence, the sport and fitness wearables need satisfy Principle 1 and Principle 3. As shown in Figure 8, the user‘s smartphone is paired with his wearables in order to analyze and process the sport and fitness data in smartphone. The user can choose the NC protocol to establish a link key for the transmission of confidential data.

#### 6.2.3. Smart Nursing

**Figure 9:**
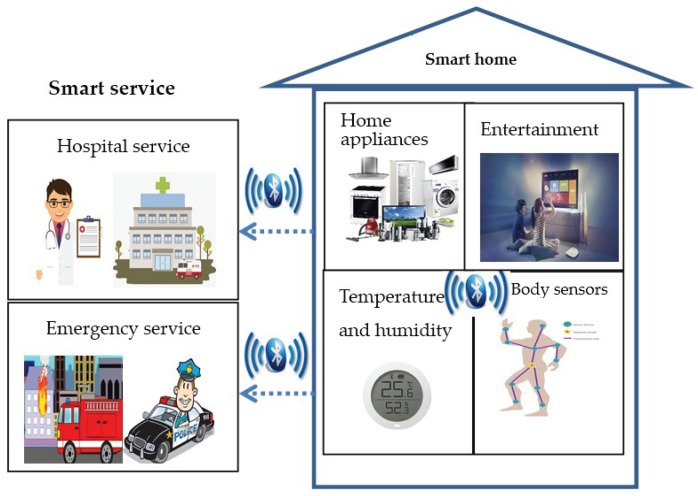
A smart nursing system.

**Figure 10:**
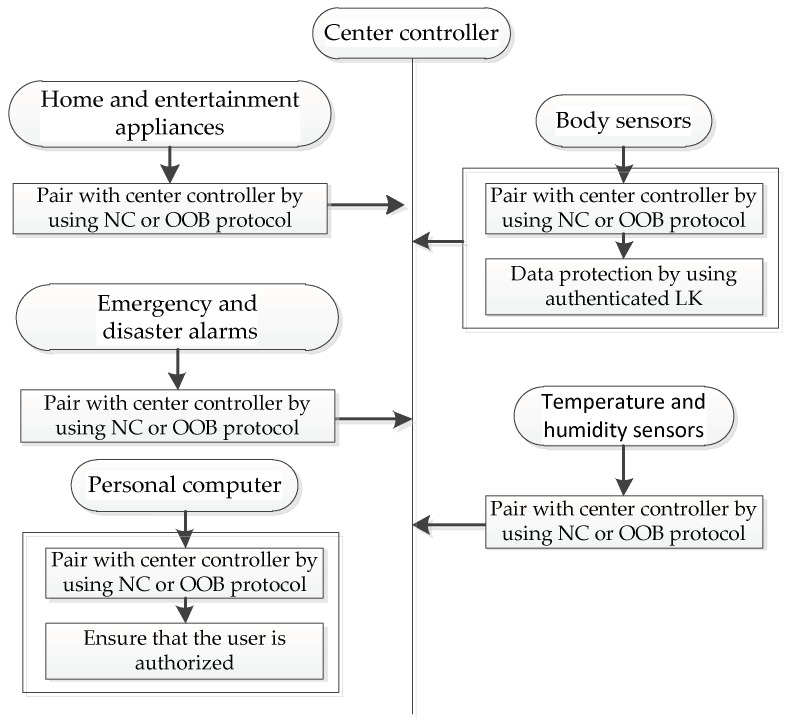
Implementation design of a Bluetooth-based smart nursing system.

As shown in Figure 9, the home appliances in the smart nursing system contain body sensors that record health data to report physical condition such as blood pressure and pulse rate, temperature and humidity detectors that assist to maintain a livable home environment, and intelligent emergency and disaster alarms that help people send rescue information in time, etc. We can see that the smart nursing system involves the transmission of private data and the access of personal authorized home appliances. Hence, this system needs to prevent malicious users from tampering commands and provide data protection. It means that Principle 1, Principle 2, and Principle 3 all fit the smart nursing system. As shown in Figure 10, various appliances choose the NC or OOB protocol to pair and bond with the center controller according to their individual needs.

## 7. Conclusions

We propose a formal security model for the SSP protocols. The NC protocol and the OOB protocol are evaluated by our security model. Our results show that both the NC protocol and the OOB protocol are secure if their cryptographic tools meet the cryptographic assumptions, i.e., the DDH assumption, the Diffie-Hellman keyed pseudorandom function, the MAC function, and the collision-resistant function. Our research confirms that the SSP solution ought to be implemented in the HAE applications. The NC protocol and the OOB protocol promise more security guarantee under the HAE environments because the security of its authenticated link key is formally analyzed under our security model.

Although the PE protocol is not analyzed, we claim that the results in Theorem 2 and Lemma 1 are also applicable to the PE protocol. However, we do not know the security result when the PE protocol plays with the active adversary. One difficulty is to formalize the passkey in the PE protocol, because the passkey is not generated by the security algorithm and is entered by the user. Formal analysis of the PE protocol is our future work.

## Figures and Tables

**Table 1:** Description of notation.

Term	Definition
Π_1_	NC protocol
Π_2_	OOB protocol
Π	Any one of Π_1_ and Π_2_
Π_i_^A, B^	Bluetooth device A’s Π_i_ instance run with Bluetooth device B, where i ∈ {1, 2}
Π^A, B^	Bluetooth device A’s any Π_1_ or Π_2_ instance run with Bluetooth device B
BD_ADDR	Bluetooth address
X or BD_ADDR_x_	BD_ADDR of Bluetooth device X
IOcapX	IO capabilities of Bluetooth device X
btlk	A predefined bit string
(SKx, PKx)	The Elliptic Curve Diffie-Hellman (ECDH) private-public key pair of Bluetooth device X
DHKey	Diffie-Hellman key
LK	Link key
Nx	Nonce (unique random value) generated by Bluetooth device X
rx	Random value generated by Bluetooth device X
Cx	Commitment generated by Bluetooth device X
Vx	Confirmation six-digit number on Bluetooth device X, which is only used in Π_1_
Ex	Check value from Bluetooth device X
ac_x_	Decision in X’s instance, where ac_x_ ∈ {true, false, *} and * denotes no decision yet made
P256()/P192()	Used to compute DHKey. If both Bluetooth devices’ hosts and controllers support secure connections, P256() is used; otherwise, P192() is used
f1()	Used to generate commitment value Cx
f2()	Used to compute LK from DHKey and random nonces
f3()	Used to generate check value Ex
g()	Used to compute six-digit numeric check value Vx
Prob(E)	Probability of event E occurring

**Table 2:** Numeric comparison (NC) protocol.

Protocol Π_1_
**Pre-protocol exchange:** Devices A and B exchange their Bluetooth addresses A and B as well as their IO capabilities IOcapA and IOcapB and a predefined bit string btlk.
**Phase 1: Public key exchange**
The initiating device A generates its own private-public key pair (SKa, PKa) and sets ac_a_ = *. Here, the private-public key pair is generated only once per device and may be computed in advance of pairing. And then, the device A sends PKa to the non-initiating device B. Similarly, the device B generates (SKb, PKb), sets ac_b_ = *, and sends PKb to the device A. The device A computes DHKey = P192(SKa, PKb) = SKa⋅PKb or P256(SKa, PKb) = SKa⋅PKb. The device B computes DHKey = P192(SKb, PKa) = SKb⋅PKa or P256(SKb, PKa) = SKb⋅PKa.
**Phase 2: Authentication stage 1 for NC**
The device A selects random Na and sets ra and rb to 0. The device B selects random Nb and sets rb and ra to 0. The device B further computes Cb = f1(PKbx, PKax, Nb, 0), where PKbx and PKax respectively denote the x-coordinate of the public keys PKb and PKa. Then, the device B sends Cb to the device A. The devices A and B exchange their Na and Nb. Upon receiving Nb, the device A checks if Cb = f1(PKbx, PKax, Nb, 0). If the check fails, the device A sets ac_a_ = false and aborts the protocol execution. The device A computes Va = g(PKax, PKbx, Na, Nb) and displays Va. Similarly, the device B computes Vb = g(PKax, PKbx, Na, Nb) and displays Vb. User checks if Va = Vb and confirms on each end. If user confirms ‘yes’, proceed the following phase; otherwise both the device A and the device B set ac_a_ = false and ac_b_ = false and terminate it, respectively.
**Phase 3: Authentication stage 2**
The device A computes Ea = f3(DHKey, Na, Nb, rb, IOcapA, A, B) and sends Ea to the device B. Upon receiving Ea, the device B checks whether Ea = f3(DHKey, Na, Nb, rb, IOcapA, A, B). If check fails, the device B must set ac_b_ = false and abort the protocol execution. Otherwise, the device B accepts the integrity of the device A and sets ac_b_ = true, computes Eb = f3(DHKey, Nb, Na, ra, IOcapB, B, A), and sends Eb to the device A. Upon receiving Eb, the device A checks whether Eb = f3(DHKey, Nb, Na, ra, IOcapB, B, A). If check fails, the device A must set ac_a_ = false and abort the protocol execution; else the device A accepts the integrity of the device B and sets ac_a_ = true.
**Phase 4: Link key calculation**
The device A computes LK = f2(DHKey, Na, Nb, ”btlk”, BD_ADDRa, BD_ADDRb). The device B computes LK = f2(DHKey, Na, Nb, ”btlk”, BD_ADDRa, BD_ADDRb).

**Table 3:** Out of band (OOB) mechanism.

Protocol Π_2_’s Phase 2
**Phase 2: Authentication stage 1 for OOB**
The device A sets ra = rand_1_ and rb = 0 and the device B also sets rb = rand_2_ and ra = 0. Here, rand_1_ and rand_2_ are random numbers. The device A computes Ca = f1(PKax, PKax, ra, 0), where PKax denotes the x-coordinate of the public key PKa. And the device B also computes Cb = f1(PKbx, PKbx, rb, 0), where PKbx denotes the x-coordinate of the public key PKb. The device A sends A, ra, and Ca to B through the human-aided OOB channel. And the device B also sends B, rb, and Cb to A through the human-aided OOB channel. Upon receiving B, rb, and Cb, the device A resets rb to the received value and if Cb ≠ f1(PKbx, PKbx, rb, 0) the device A sets ac_a_ = false and aborts the protocol execution. If step 3 received and B’s IO capability does not indicate OOB authentication data present set, set ra = 0. Upon receiving A, ra, and Ca, the device B resets ra to the received value and if Ca ≠ f1(PKax, PKax, ra, 0) the device B sets ac_b_ = false and aborts the protocol execution. If step 3 received and A’s IO capability does not indicate OOB authentication data present set, set rb = 0. The device A selects random Na and the device B selects random Nb. The devices A and B exchange Na and Nb to each other.

**Table 4:** Security properties comparison among related protocols.

Protocol	Passive Eavesdropping Attack	MITM Attack
Our NC protocol	Yes	Yes
Our OOB protocol	Yes	Yes
Yeh et al. [23]	No	Yes
Gajbhiye et al. [12]	Yes	Yes
Gajbhiye et al. [13]	Yes	Yes(except for the JW model)

**Table 5:** Experimental parameters and algorithms.

Parameter	Cryptographic Algorithm	Description
PKx and SKx	ECDH_Key	ECDH key-pair generation
DHKey	P256() ^1^	DHKey computation
Nx	Rand	128-bit pseudo-random number generator
Cx	HMAC-SHA256	SHA-256-based MAC
Vx	SHA-256	Cryptographic hash algorithm
Ex	HMAC-SHA256	SHA-256-based MAC
LK	HMAC-SHA256	SHA-256-based MAC
Signature ^2^	HMAC-SHA256	SHA-256-based MAC
XOR value ^3^	XOR	Exclusive or
Encrypted information ^4^	AES-256_Enc	Advanced encryption standard (AES) encryption algorithm with 256-bit key
IO capability and other information ^4^	AES-256_Dec	AES decryption algorithm with 256-bit key

^1^ P192() and P256() are optional and we take P256() for an example here. ^2^ Signature is only used in the enhanced NC protocol proposed by Gajbhiye et al. [12]. ^3^ XOR is only used in the improved NC protocol proposed by Yeh et al. [23]. ^4^ Encrypted information/IO capability and other information are only used in the SSP protocol with delayed-encrypted IO (SSP-DEIO) proposed by Gajbhiye et al. [13].

**Table 6:** Computation cost of each protocol.

Protocol	Computation Cost
Our NC protocol	2ECDH_Key + 2P256() + 2Rand + 8HMAC-SHA256 + 2SHA-256
Our OOB protocol	2ECDH_Key + 2P256() + 2Rand + 10HMAC-SHA256
Yeh et al. [23]	2ECDH_Key + 2P256() + 4XOR + 6HMAC-SHA256
Gajbhiye et al. [12]	4ECDH_Key + 4P256() + 2Rand + 6HMAC-SHA256
Gajbhiye et al. [13]	4ECDH_Key + 4P256() + 2Rand + 8HMAC-SHA256 + 2SHA-256 + 2AES-256_Enc + 2AES-256_Dec
